# Episodic Autobiographical Memory in Adults With Autism Spectrum Disorder: An Exploration With the Autobiographical Interview

**DOI:** 10.3389/fpsyt.2020.593855

**Published:** 2021-02-01

**Authors:** Romain Coutelle, Marc-André Goltzene, Marie Canton, Mélodie Campiglia-Sabourin, Juliette Rabot, Éric Bizet, Marie Schoenberger, Fabrice Berna, Jean-Marie Danion

**Affiliations:** ^1^Department of Psychiatry, Hôpitaux Universitaires de Strasbourg, University of Strasbourg, Strasbourg, France; ^2^Service de pathologie professionnelle et environnementale, Hôpitaux Universitaires de Strasbourg, Strasbourg, France; ^3^Unité de Neuropédiatrie, Centre référent pour les troubles du langage et des apprentissages (CLAP), Hôpital d'Enfants, CHU Brabois, Vandoeuvre-Lès-Nancy, France; ^4^Centre hospitalier spécialisé de Rouffach, Centre Ressources Autisme Alsace, Rouffach, France; ^5^UFR de psychologie, Université de Strasbourg, Strasbourg, France; ^6^Centre Psychothérapique de Nancy, Centre Ressources Autisme de Lorraine, Nancy, France

**Keywords:** autism spectrum disorder, episodicity, autobiographical memory, autobiographical interview, cueing

## Abstract

**Introduction:** The literature has provided contradictory results regarding the status of episodic memory in autism spectrum disorder (ASD). This might be explained by methodological differences across studies. In the present one, the well-recommended Autobiographical Interview was used in which important aspects of episodic memory were assessed, namely, the number and richness of phenomenological memory details, before and after a retrieval support.

**Method:** Twenty-five well-documented adults with ASD without Intellectual Disability (nine women) and 25 control participants were included and asked to recall six specific autobiographical events. The number and richness of details were assessed globally and for five categories of details (perceptual/sensory, temporal, contextual, emotional, and cognitive), firstly before and then after a specific cueing phase consisting in a series of specific questions to elicit more precise memory details.

**Results:** Cumulatively, from the spontaneous recall to the cueing phase, the number of internal details was lower in ASD individuals compared to controls, but this difference was relevant only after the specific cueing procedure and observed only for contextual details. In contrast, no relevant group difference was observed during spontaneous recall. The detail richness was not impaired in ASD throughout the Autobiographical Interview procedure.

**Conclusion:** Our results speak against a clear impairment of episodicity of autobiographical memory in ASD individuals. They thus challenge previous ones showing both a reduced specificity and episodicity of autobiographical memory in this population and call for further studies to get a better understanding on the status of episodic autobiographical memory in ASD.

## Introduction

Episodic autobiographical memories consist in episodic elements—that is, summary records of experience often in the form of visual images associated to sensory–perceptual–cognitive–affective information—that are embedded in a more complex conceptual frame consisting in semantic autobiographical knowledge (1). Episodic memories are associated to a specific state of consciousness during remembering called conscious recollection in which the person mentally relieves a past event and feels in the present the past feelings related to it. This mental time travel is therefore crucial to provide a sense of self-continuity in time, which is the hallmark of the experiential component of self, namely, the “I self” (2, 3). Investigating episodic autobiographical memories is consequently particularly interesting to appreciate the nature of the intimate relationship between the self and autobiographical memory. Classical measures of episodic memories include the counting of memory details and evaluation of the details' richness, the latter reflecting the strength of the feeling of reliving associated with remembering (4).

Autism spectrum disorder (ASD) is a neurodevelopmental disorder that manifests itself through socio-communicational deficits on the one hand and repetitive patterns and restrictive interests on the other (5). Although ASD is characterized by difficulties in social relationships, specific competences linked to restricted interests have been underlined by testimonies (6), autobiographies (7, 8), and researches (9, 10). Several studies including individuals with ASD have reported greater difficulty to retrieve specific memories in both adults (11–21) and children (15, 22–24). Studies in adults with ASD showed less specific autobiographical memories (11–15, 17–21) in this population compared to control participants with the exception of Crane et al.'s study (25). Other studies reported greater difficulties for adults with ASD to access past events as reflected by the longer time for them to retrieve those events (11, 12, 14, 26). Although these studies pointed to an alteration of the episodic component of autobiographical memory in adults with ASD, the studies that have assessed the component of episodicity, namely, memory details and state of consciousness, have led to contradictory results. For instance, in Lind et al.'s (20) study, the number of memory details (spatial references, entities present, sensory descriptions, thoughts/emotions/actions, and temporal differences) rated by the experimenter did not differ between ASD and controls but two of the four subscales were characterized by a floor effect that may have masked existing differences. In contrast, the details richness was lower in the ASD group. Another study about early memories, using online self-reports, compared 83 adults with ASD to controls (27). The authors showed that autobiographical memories in the ASD group were richer in sensory details and poorer in social details than in the control group. Last, regarding the state of consciousness associated with remembering, one study found an impaired autonoetic consciousness in ASD (18), while Lind did not replicate this result (19). All in all, in contrast to the well-documented reduced memory specificity in ASD, results on episodicity in autobiographical memory remain contradictory.

Among scales and instruments to measure autobiographical memory episodicity, the Autobiographical Interview (AI) (4) is today the most developed and the most precise one. It has been already used in healthy young and elderly adults (4, 28), in patients with parietal lesions (29), schizophrenia (30), mild cognitive impairment (31), depression (32), or PTSD (33). It is designed specifically to assess both the number and richness of phenomenological memory details before and after a retrieval support. The procedure comprises three recall steps, namely, spontaneous recall (SR), general cueing, and specific cueing (SC) of memories, the latter using specific questions to retrieve details related to predefined categories. By doing so, it makes it possible to assess both the spontaneous access to memory details and those details that have been stored but become accessible only after external aid. This method is therefore particularly suitable for a thoughtful examination of episodic autobiographical memory. We considered it highly relevant to further investigate the memory in ASD and used it on a larger sample of adults with a well-documented diagnosis of ASD without ID.

Given the contradictory results found in the literature reviewed above, the aim of the present study was to investigate the characteristics of episodic autobiographical memories in adults with ASD without ID. Thanks to the use of Levine's procedure, we could make the following predictions: a reduced number or richness of memory details at both SR and SC phase would plead for an impaired episodic memory and point to a deficient memory encoding; an increase of memory details leading to a normalization of performance in the ASD group during SC would argue for a deficient retrieval processes involved in episodic autobiographical memory impairment in ASD. On the contrary, an absence of group difference or even an increase of memory details in the ASD group would speak against a deficient episodic autobiographical memory in ASD. Last, to take into account the clinical profile of ASD, memories were additionally cued according to restricted interests, social relationships and holidays in order to examine the influence of memory themes on memory details.

## Methods

### Participants

Twenty-five individuals with ASD without ID participated in the study (16 men and 9 women). They have been recruited in three French expert centers for diagnosis and evaluation of ASD in Nancy, Colmar, and Strasbourg. All the individuals had obtained an established diagnosis of ASD in these centers. ASD individuals with a history of traumatic brain injury and alcohol and substance abuse were not included. Those with a current clinical diagnosis of Major Depressive Disorder and with IQ lower than 70 were not included either.

The comparison group consisted of 25 participants (16 men and 9 women) matched to the patient group in terms of age, gender, and level of schooling. None of them had medical illness or history of mental illness. Participants with clinical depression or IQ lower than 70 were not included.

This study was approved by the Strasbourg Ethics Committee (“Comité de protection des personnes-Est-IV”), N°16/07. After complete description of the study to participants, written informed consent was obtained. All the participants who took part in the study received a financial compensation.

### Procedure

All the individuals had previously completed a standardized and pluridisciplinary assessment following French recommendation for diagnosis of ASD in adults (34). During this initial evaluation, the standard diagnosis tools used were ADI-R (35) and/or ADOS module 4 [version 1 (36) and 2 (37)]. At the time of the study, participants were assessed for depression [using the BDI scale (38, 39)] and DSM-5 (5) criteria for Major Depressive Disorder (in case of BDI score above eight), intellectual efficiency [WAIS-III short form (40) validated in French (41)], and self-esteem [the Rosenberg Self-Esteem Scale (42) validated in French (43)]. Then, a neuropsychological assessment including verbal fluency, mental flexibility, and inhibition was included followed by an autobiographical memory task.

### Materials

#### Questionnaires Assessing Autistic Symptoms

The Autism Spectrum Quotient (AQ) (44), validated in French (45), is a usual and rapid screening instrument for ASD. Participants completed this auto-questionnaire of 50 items divided into five 10-item subscales: social skills, attention-shifting, attention to details, communication, and imagination.

The Ritvo Asperger Autism Rating Scale-Revised (RAADS-R) (46, 47) is an 80-item self-report instrument, currently validated in French, to assist the diagnosis of adults with ASD. The scale comprises four symptom domains (social relatedness, language, sensorimotor, and circumscribed interests), which are in line with the current conceptualization of ADS [DSM-5 (5)] distinguishing, on the one hand, socio-communicational deficits (social relatedness and language) and, on the other hand, restrictive and repetitive patterns (sensorimotor and circumscribed interests).

Last, the Empathy Quotient (EQ) (48), validated and translated in French (45), is a short 40-item self-administered questionnaire tapping empathy. It also includes 20 filler items.

#### Neuropsychological Evaluation

We selected three tasks to assess the severity of frontal/executive dysfunctions of our patients: verbal fluency (49) was assessed using both a semantic (animals) and a phonological (French words starting with P) fluency task of 60 s each; inhibition was assessed using the French adaptation of the Hayling test (50); and mental flexibility was assessed using the Trail Making Test (51) Parts A and B, by subtracting connecting time in part A from connecting time in part B.

#### Autobiographical Memory Task

##### Spontaneous Recall

Participants had to spontaneously recall and tell six positive or negative autobiographical events that were important for them, located in time and place, and had lasted <24 h. Three types of themes were explored: interests, social relationships, and holidays. In memories related to holidays, interest and social topics had not to be at the forefront. This was explicitly mentioned to all participants. The themes social relationships and interests were chosen to echo to the dyadic description of ASD diagnosis in DSM-5, which combines persistent deficits in social communication and social interaction (cue word social relationships) and restricted, repetitive patterns of behavior, interests, or activities (cue word interests). A neutral condition was added. The theme “holidays” was selected because it was not a priori related to ASD symptomatology. Other themes such as “family” (that are classically used as cue word in autobiographical memory tests) were not suitable because of social relationship involvement.

The “current year” life period was not explored to avoid any ceiling effect due to the recency of memories. So, we asked participants to tell memories older than 1 year. Then, participants were asked to recall two events from each theme. The order of memory recall was randomized for each participant. No amount of time was allocated to produce each memory.

Then, participants gave each memory a title in the form of a keyword or short phrase summing up the event. They were asked to remember each title so that if it was given back to them, they would be able to recall to which memory it corresponded. The time needed to complete the AM recall task during this SR phase was around 30 min (5 min per event).

##### SC of Memories

The cueing method used was adapted from the AI (4). Contrary to previous studies using this method (4, 30), we did not use “general cueing.” In these past procedures, after each spontaneous memory recall, participants were given a general cue that simply clarified the instructions and encouraged them to add some information to their memories (e.g., Do you have any other details to add?). As general cueing recall did not differ from SR in these studies, we did not maintain this cueing.

Then, the SC was applied after all six memories had been recalled in order to avoid any influence of the specific questions on the subsequent memories. It consisted in asking participants several specific questions following the procedure of Levine's manual (4) to elicit all these details. In practical terms, each memory title was given to the participants orally and they were asked to answer specific questions to give memory details. Following previous adaptations of AI by Potheegadoo, the specific questions were divided into five categories of details: perceptual/sensory (e.g., Can you describe the sounds in your memory?); temporal (e.g., Do you remember the year it happened?); contextual (e.g., Can you describe other persons or objects around you at the time of the event?); emotional (e.g., How did you feel when this event happened?); and cognitive (e.g., Do you remember the thoughts you had at that very moment?). These categories were rearrangement of Levine's categories labeled “perceptual,” “time,” “event,” “place,” and “emotional/thought” (4). So, the contextual category embraced event and place when the emotional and thought categories corresponded to emotional/thought splitting. It took around 1 1/2 h to complete the SC phase, that is, 15 min per memory.

#### Memory Scoring

All memories were audio-recorded and transcribed for scoring and analysis. The overall memory specificity and the number and richness of phenomenological details were assessed for both phases by two independent raters on 10% of the memories obtained. The two independent raters were MC and MCS. They both are neuropsychologists with an expertise in neurodevelopmental disorders. They had also received training in AM. They were blind to the status of the subject. The studied events were selected at random. Disagreements were discussed between raters until a consensus was found.

##### Memory Episodicity

Memories were scored individually on a four-point scale (52). There was a substantial agreement between the raters during both the SR (Cronbach's α = 0.89) and SC phases (Cronbach's α > 0.99). This last agreement was very high. In fact, as the procedure used during the SC phase elicited many details about time, place, feelings, thoughts, perceptions, and what was occurring during the event, the score was, by construct, maximal (score of 4) in almost all the memories when cumulating SR and SC phase.

##### Number of Phenomenological Details

The number of details was counted using the AI method previously and extensively explained in Levine's initial and seminal article (4). As recommended by Levine (4), we distinguished internal from external details. Internal details were those that pertained directly to the main event described by the participant, were specific to time and place, and were considered to reflect episodic re-experiencing. They were classified into five categories of details (perceptual/sensory, temporal, contextual, emotional, or cognitive). External details referred to semantic information, repetitions, and details from other incidents external to the main event recalled. Finally, external details also included other details such as metacognitive statements. We mostly focused on internal details. Thus, external details had been studied not separately but only as a whole. There was a high degree of reliability between raters (SR: Cronbach's α = 0.98; SC: Cronbach's α = 0.95).

In addition, as recommended by Levine (4), scoring was done separately for each condition (SR, SC), but scores were also analyzed cumulatively across both levels of recall. That is, SC details were added to details generated from the SR prior condition. This scoring method followed the natural discursive tendency for participants to assume that information given in earlier conditions was implicit in later conditions (i.e., participants did not repeat information they had already given).

##### Detail Richness

The richness of perceptual/sensory, temporal, contextual, emotional, and cognitive details as well as the global details richness of each memory was assessed on a three-point scale (from 0 to 3) (4). Here, again, there was a high degree of reliability between raters on 10% of the memories obtained (SR: Cronbach's α = 0.89; SC: Cronbach's α = 0.90).

#### Self-Ratings of the Memories

##### Subjective State of Consciousness

After the SR phase, the participant was asked to determine the subjective state of conscious awareness associated with memory retrieval using the Remember/Know/Guess Procedure (53, 54). This is a first-person procedure for assessing subjective states of conscious awareness associated with the recall of a memory. Participants were instructed to give a Remember, Know, or Guess response according to whether the retrieved memory was associated with conscious recollection, familiarity, or guessing, respectively. A Remember response was defined as the mental ability to re-live specific aspects such as perceptions, thoughts, or feelings experienced at the time of the event. A Know response was defined as simply knowing what happened, in the absence of any conscious recollection. A Guess response corresponded to aspects of the events that were neither consciously recollected nor simply known, but guessed. Participants were given instructions and examples of each kind of response before the test.

##### Visual Perspective

After the SR phase, the participant was invited to describe his/her visual perspective during remembering (55). Did he/she see him/herself in the scene as an external observer would have seen it (observer perspective)? Did he/she see the scene from his/her own point of view (field perspective)?

#### Data Analysis

Data are presented as means and standard deviations for continuous variables and percentages for categorical data.

In all analyses, the probability that the score of each measure was higher in the control group than in the ASD group was calculated. A probability higher than 95% or lower than 5% were both considered meaningful. It is worth reminding that a probability of 5% that B is greater than A is equivalent to a probability of 95% that A is greater than B.

To carry out the analyses, we used Bayesian techniques (56). We compared the variables of interest between the two groups on the entire sample, using normal distributions for continuous variables and beta distributions for binomial data. Univariate subgroup comparisons according to the theme of memories were also made, using the same methods.

For the comparison of memory variables, hierarchical regression models were used. These models were linear for quantitative variables and logistic for qualitative variables. A random effect per subject was placed on the intercept due to repeated data in the same subject. The analyses were supplemented by multivariate hierarchical regression models for adjustment on IQ and depression score (IDB). Correlation coefficients were calculated using a two-dimensional multinormal distribution.

To appreciate the differences between the two groups, the probability for the difference to be >0 or the probability for the relative risk to be >1 was calculated.

In order to perform the Monte Carlo integration, we used three Markov chains with 32,000 iterations each, a 2000-iteration burn-in, and a thinning of three. The convergence was checked graphically and with the potential scale reduction factor.

The analyses were done using the software R version 3.4.3 R Core Team (57).

## Results

### Clinical and Neuropsychological Evaluation

Scores of both individuals with ASD and controls are presented in Table 1. The IQ scores of ASD individuals were higher than those of controls. ASD individuals also had higher level of depressive symptoms and lower scores of self-esteem than controls.

**Table 1:** Clinical and neuropsychological data of individuals with ASD and comparison participants.

	**Comparison** ** participants ** **(gp 1)** ** (*****n*** **=** **25)**		**Individuals** ** with ASD** ** without ID** ** (gp 2)** ** (*****n*** **=** **25)**		**Probability** ** group 1 >** ** group 2**
	**Mean**	**(SD)**	**Mean**	**(SD)**	
Age (years)	31.84	(11.75)	30.68	(10.30)	0.63
Sex (men) (*N*, %)	16	(0.64)	16	(0.64)	0.5
IQ	97.32	(10.87)	110.12	(17.49)	<0.01^*^
**Years of schooling (*****N*****, %)**					
<9 years	1	0.04	1	0.04	0.5
9 to 11 years	4	0.16	4	0.16	0.5
12 to 13 years	1	0.04	1	0.04	0.5
14 years	9	0.36	9	0.36	0.5
15 to 16 years	5	0.20	5	0.20	0.5
More than 16 years	5	0.20	5	0.20	0.5
**ADI-R^a^**					
Social			14.92	(4.33)	
Communication			8.08	(4.03)	
Restrictive, repetitive			4.69	(1.43)	
behavior					
ADOS^b^ module 4 (Communication and social behavior)			9.00	(3.19)	
ADOS-2^c^ module 4 (Communication and social behavior)			11.17	(5.57)	
AQ^d^ (total)	12.52	(5.52)	34.28	(7.57)	<0.01^*^
Social skills	1.48	(2.33)	7.84	(2.30)	<0.01^*^
Attention-shifting	3.52	(2.02)	8.08	(1.38)	<0.01^*^
Attention to details	4.92	(2.27)	6.64	(2.12)	<0.01^*^
Communication	1.16	(1.66)	6.68	(2.41)	<0.01^*^
Imagination	2.00	(1.44)	5.20	(2.08)	<0.01^*^
EQ^e^ (total score)	37.88	(7.99)	22.36	(8.30)	>0.99^*^
RAADS^f^ (score total)	47.88	(24.49)	143.32	(34.75)	<0.01^*^
Social relatedness	17.76	(10.24)	68.68	(19.25)	<0.01^*^
Circumscribed interests	11.64	(6.79)	29.52	(7.84)	<0.01^*^
Language	3.92	(2.18)	9.64	(4.45)	<0.01^*^
Sensorimotor	14.56	(10.54)	35.60	(13.48)	<0.01^*^
Self-esteem	50.88	(7.09)	39.08	(8.41)	>0.99^*^
BDI^g^ (total score)	2.20	(3.29)	7.68	(5.91)	<0.01^*^
TMT^g^ (mental flexibility score)	30.38	(23.29)	30.76	(29.09)	0.49
Hayling test (inhibition score)	4216	(2042)	4283	(1914)	0.50
Phonologic fluency	14.50	(3.41)	15.00	(4.44)	0.33
Semantic fluency	21.33	(5.22)	20.52	(6.43)	0.69

a *Autism Diagnosis Interview, Revised form*.

b *Autism Observation Schedule, version 1*.

c *Autism Observation Schedule, version 2*.

d *Autism Quotient*.

e *Empathy Quotient*.

f *Rivto Autism Asperger Diagnosis Scale*.

g *Beck Depression Inventory*.

*^h^Trail Making Test*.

* *Probability higher than 95% or lower than 5% indicating a relevant difference between groups*.

The total scores and the majority of the subscores of AQ, RAADS, and EQ strongly differed between the ASD and the comparison groups. Only the attention to details subscore of AQ did not differ between the two groups. Finally, executive functions did not differ between the two groups.

### Number and Richness of Memory Details at SR and SC Phases

At the SR phase (Table 2), the number and the richness of details did not differ between groups. This result was influenced neither by memory theme (interests, social relationships, and holidays) nor by gender. To assess the potential effect of the difference of IQ and BDI score between the groups, we conducted complementary analyses. Mixed regression analysis showed that the effect of IQ did not differ on both groups (β = 0.002, 95% CI [−0.05; 0.05], *P*(β > 0) = 0.47). Another mixed regression analysis also evidenced that the effect of BDI score on those groups was not different (β = 0.61, 95% CI [−0.51; 1.71], *P*(β > 0) = 0.86).

**Table 2:** Overall memory episodicity, number, richness of memory details for spontaneous recall (SR), specific cueing (SC) phases, and cumulative SR + SC phases obtained from SC in individuals with ASD without ID and comparison participants.

		**Comparison participants (gp 1) (*****n*** **=** **25)**		**Individuals with ASD without ID (gp 2) (*****n*** **=** **25)**		**Probability group 1 > group 2**
		**Mean**	**(SD)**	**Mean**	**(SD)**	
Overall memory episodicity	SR	2.79	(1.04)	2.67	(0.95)	0.56
	SC	3.98	(0.25)	3.92	(0.40)	0.57
Number of details	SR	14.14	(15.55)	12.05	(7.70)	0.82
	SC	39.05	(14.75)	33.41	(14.86)	0.98^*^
	SR + SC	53.22	(25.81)	45.59	(19.24)	0.99^*^
Richness of details	SR	1.22	(0.57)	1.19	(0.57)	0.55
	SR + SC	2.56	(0.54)	2.43	(0.54)	0.60

* *Probability higher than 95% or lower than 5% indicating a relevant difference between groups*.

After the SR and the SC phases (Table 2), the memories of individuals with ASD had lower internal details than those of control participants when external details were similar. The richness of details did not differ between the two groups. These results were not influenced by memory theme, gender, or IQ.

At the SR phase, the number and richness of all detail categories did not differ between groups whereas the number of contextual details was found lower in ASD individuals after the SR and the SC phases (Table 3). These results were influenced neither by memory theme nor by gender.

**Table 3:** Number and richness of phenomenological details of memories per detail category for spontaneous recall (SR) and specific cueing (SC) phases in individuals with ASD without ID and comparison participants.

**Details category**			**Comparison** ** participants (gp 1)** ** (*****n*** **=** **25)**		**Individuals with ASD** ** without ID (gp 2)** ** (*****n*** **=** **25)**		**Probability group** ** 1 > group 2**
			**Mean**	**(SD)**	**Mean**	**(SD)**	
Perceptual/sensory	Number	SR	1.25	(2.63)	1.128	(1.58)	0.55
		SC	12.32	(5.78)	11.91	(6.18)	0.45
		SR + SC	13.59	(7.27)	13.05	(6.88)	0.63
	Richness	SR	0.70	(1.05)	0.85	(1.06)	0.45
		SR+SC	2.97	(0.27)	2.95	(0.30)	0.55
Temporal	Number	SR	1.75	(1.58)	1.44	(1.39)	0.62
		SC	5.28	(1.60)	4.55	(1.72)	0.53
		SR+SC	7.04	(2.36)	6.01	(2.37)	0.78
	Richness	SR	0.99	(0.92)	0.95	(0.75)	0.53
		SR + SC	2.88	(0.46)	2.66	(0.60)	0.63
Contextual	Number	SR	9.38	(11.86)	7.71	(5.31)	0.82
		SC	15.74	(9.34)	11.65	(8.19)	0.99^*^
		SR + SC	25.14	(18.12)	19.45	(11.21)	>0.99^*^
	Richness	SR	2.69	(0.62)	2.67	(0.61)	0.55
		SR+SC	2.94	(0.26)	2.88	(0.32)	0.57
Emotional	Number	SR	0.97	(1.26)	1.05	(1.50)	0.53
		SC	3.15	(1.98)	2.86	(2.08)	0.55
		SR+SC	4.11	(2.62)	3.92	(2.92)	0.61
	Richness	SR	0.84	(0.98)	0.84	(0.96)	0.55
		SR+SC	2.23	(0.90)	1.99	(0.91)	0.63
Cognitive	Number	SR	0.78	(1.70)	0.72	(1.22)	0.54
		SC	2.56	(2.39)	2.44	(2.63)	0.52
		SR+SC	3.33	(3.28)	3.17	(3.23)	0.56
	Richness	SR	0.59	(0.95)	0.61	(0.93)	0.52
		SR + SC	1.78	(1.08)	1.60	(1.20)	0.52

* *Probability higher than 95% or lower than 5% indicating a relevant difference between groups*.

Correlation analysis in patients found one relevant correlation between inhibition and the details richness during the SR phase (*r* = −0.32; 95% CI: [−0.64; −0.06]; *Pr* (*r* > 0) = 0.049) meaning that richness was higher in ASD individuals with better inhibition performances. Correlations with other cognitive measures and autobiographical performance were not relevant. In controls, the total number of details and that of richness during the SR phase were both positively correlated with lexical verbal fluency [*r*s > 0.31; *Pr*s (*r* > 0) > 0.95].

### Self-Ratings of Memories

The subjective state of conscious awareness associated with memory retrieval and the visual perspective did not differ between both groups (data not shown).

## Discussion

The aim of the present study was to investigate the characteristics of episodic autobiographical memories in adults with ASD without ID. Our results showed that when participants were asked to spontaneously narrate their memories, neither the number of details nor their richness differed between the ASD and the control groups. Following the use of a SC procedure, the gain in internal details was lower in ASD, this being due to the fact that less contextual details were added. As a consequence, the number of internal details, particularly contextual details that were collected after SR and SC, was lower in ASD. Analyses on the other categories of details did not show differences between groups. Finally, neither the subjective state of consciousness nor the visual perspective associated with memory retrieval differed between groups. This coherent pattern of results speaks against a clear impairment of episodic autobiographical memories and adds further data to the controversial literature on this topic.

During the first phase of SR, our results did not show any episodic autobiographical memory deficit in ASD individuals. In contrast, two studies (18, 20) argued for an impaired episodic autobiographical memory in this population by reporting a reduced details richness and/or an impaired autonoetic consciousness associated with memory retrieval. Methodological differences may account for the discrepancy observed with our study. In the Tanweer et al. study (18), the sample of ASD participants was small (*n* = 11), the diagnosis was not confirmed with standard clinical instrument, and groups were not matched on gender. Most of all, autobiographical memories were cued on lifetime periods (18) or on pictures [such as beach, museum, or pub (20)] that were not personally relevant, while we cued the memories on social relationships and interests that might be especially self-relevant in ASD and facilitate the access to memory details. In fact, the self-relevant properties of interests have been highlighted by their pervasive characteristics in daily life (58, 59) and the major positive values that adults with ASD ascribe to them (10). The self-relevant properties of social relationships may seem counterintuitive. However, social relationships are main issues in ASD and one study (60) showed that ASD participants quoted their autobiographical memories as Self-Defining Memories more often than did the controls. It might be opposed that this assumption of self-relevant cues does not explain why, in our study, episodicity was not impaired in memories cued on holidays, viewed as the neutral condition. To this regard, we checked the content of the memories cued in ASD individuals. Their main theme was in fact related to the corresponding cue words only for the cueing on interest and social relationships. Several memories cued on holidays also involved social relationships (43,88%, data not shown). Thus, the cue word “holidays” was not as neutral as it was supposed to be. Further studies are therefore needed using a better neutral condition to verify whether the particular self-relevance of our memories was a critical factor accounting for the absence of group difference observed in episodicity [see also Crane et al.'s 2010 study (13) on this point].

Our study is not the first to challenge the issue of an episodic autobiographical memory deficit in ASD individuals. In fact, one study (27) investigating early childhood memories even reported that ASD individuals included more sensory details and more detailed descriptions of things in their memories compared to controls. As the details referred mostly to sensory input and objects we can infer, as the authors did, that, in the ASD group, the first memories relied mostly on circumscribed interests. This tropism for non-social topics linked to circumscribed interests is, indeed, well-shown during infancy in ASD (61). We did not observe more sensoriperceptual details in our study, but the memories we investigated were not limited to those of early childhood and included more recent life experiences. Furthermore, due to the web-based design, the diagnostic status of ASD individuals could not be checked by means of face-to-face interview, thus raising concerns about the validity of the ASD diagnosis. Future studies would therefore gain in investigating early childhood memories in people clinically diagnosed with ASD and using Levine's method of details counting in order to investigate whether better skills in episodic memory are in fact observed in ASD individuals than in controls.

During the SC phase, adults with ASD added less contextual details than controls did. The contextual category embraces distinct subtypes of details such as narrator's actions, other protagonists involved in the event, or places. Since those details were slightly weaker in the ASD group during the first phase of retrieval, SC procedure has probably enhanced the difference between both groups for one of these detail subtypes. To test this hypothesis, we conducted a secondary analysis of details related to others and details related to places. Although location details did not differ between both groups [*Pr*(Ctrl > ASD) = 0.85, data not shown], social details were lower in ASD [Pr(Ctrl > ASD) > 0.99, data not shown]. This result aligns with those of previous studies showing that early childhood memories had less social details and less words referring to the social in-group (21, 27). All in all, these results argue for a careful attention toward social details in future studies investigating episodic autobiographical memories in ASD. In fact, this very specific detail category may, in some case, account for an impaired episodicity in ASD (as it was in our study for one measure of episodicity).

The investigation of the classical determinants of autobiographical memory such as gender and executive functions led to two main results. First, gender did not influence the results on number and richness of details whatever the phase considered. Our results about gender contrast with those of Goddard et al.'s (62) in children with ASD. Females in both the typical development and ASD groups generated more detailed and emotional memories than males (62). Thus, these authors highlighted how a deficient capacity to retrieve specific memories was more characteristic of male participants in the ASD group. We did not confirm this result in our population. The second result was the correlation between inhibition and the details richness during the spontaneous phase. The link between executive functions and autobiographical memory performances has been previously shown in typical adults (63), in recovered depressed women (64), in adults with schizophrenia (65), and in children with ASD without ID (66). Thus, our study confirms and extends the past results obtained in children with ASD.

From the SR phase to the SC phase, the exploration of phenomenological characteristics of memories such as number of details, richness, state of consciousness during retrieval, and visual perspective did not show a clear impairment in ASD individuals. Number of details, richness, and state of consciousness have been discussed earlier. Past studies about visual perspective led to contradictory results in adults with ASD. One study reported more frequent observer perspective in the ASD group compared to controls (19) while another did not (26). Thus, the results about phenomenological characteristics of autobiographical memories in ASD suggest that episodic autobiographical memory performances might be close to standard population attainments. A recent review has shown that neurocognitive differences between individuals with and without ASD have decreased in studies over time in the last decades (67). The authors hypothesized that this decrease may have occurred because of changes in the definition of autism from a narrowly defined and homogeneous population toward an inclusive and heterogeneous population. Such a trend might impact the results about episodicity in adults with ASD through time from a clear discrepancy to a tiny or no difference. Finally, although our results suggest an absence of episodic memory deficit, this does not mean that the functions of autobiographical memory (i.e., the way memories are used independently to the access to memory details themselves) are spared (68). In fact, two studies from our lab showed that social function of autobiographical memory was impacted (69, 70) so that people with ASD less frequently used their autobiographical memory in order to interact with others; interestingly, ASD individuals did not differ from controls in their use of their memory for better understanding themselves or to project themselves in the future. Thus, episodic impairment in ASD might be more functional than structural. New research is needed to test this assumption.

We have to acknowledge several limitations of our study. We have already underlined the unexpected group difference of IQ, thus raising the issue of the impact of IQ in our results. However, complementary analyses showed that the influence of IQ on episodicity did not differ between groups, thus ruling out the hypothesis that higher IQ in ASD have masked group differences with our control group. Another limitation concerns the modification of Levine's protocol we did. We cued the memories on three themes (social relationships, interests, and holidays) when, in Levine's study, memories were cued on life periods. Finally, as hypothesized earlier, these modifications may have increased the episodicity of the autobiographical memories retrieved.

Keeping in mind that an absence of difference should not be interpreted as an evidence for a non-existing difference, our results did not find a clear reduced episodicity of autobiographical memory in a sample of ASD individuals. They thus challenge previous ones showing a deficit of autobiographical memory in this population and call for further studies to get a better understanding on the status of episodic autobiographical memory in ASD.

## Data Availability Statement

The datasets presented in this article are not readily available because the datasets generated and/or analyzed during the current study are not publicly available due to French laws about data protection. Requests to access the datasets should be directed to Romain Coutelle, romain.coutelle@chru-strasbourg.fr.

## Ethics Statement

The studies involving human participants were reviewed and approved by Strasbourg Ethic Committee (Comité de protection des personnes-Est-IV), N°16/07. The patients/participants provided their written informed consent to participate in this study.

## Author Contributions

J-MD and RC designed the study. MC, MC-S, EB, and RC collected the data. M-AG performed the statistical analyses. RC wrote the first complete draft of the manuscript. J-MD and FB provided substantial modification to the manuscript. All authors read and approved the final manuscript.

## Conflict of Interest

FB has received a speaker honorarium from Astra Zeneca, Lundbeck, Janssen-Cilag, and Bristo-Meyers-Squibb. The remaining authors declare that the research was conducted in the absence of any commercial or financial relationships that could be construed as a potential conflict of interest.

## Abbreviations

ADI-R: autism diagnosis interview, revised form
ADOS-1: autism observation schedule, version 1
ADOS-2: autism observation schedule, version 2
AM: autobiographical memory
AQ: autism quotient
ASD: autism spectrum disorder
BDI: beck depression inventory
EQ: empathy quotient
ID: intellectual disability
IQ: intelligence quotient
RAADS: ritvo autism asperger diagnosis scale
TD: typical development
TMT: trail making test
SR: spontaneous recall
SC: specific cueing.
